# Can interbreeding of wild and artificially propagated animals be prevented by using broodstock selected for a divergent life history?

**DOI:** 10.1111/j.1752-4571.2012.00247.x

**Published:** 2012-11

**Authors:** Todd R Seamons, Lorenz Hauser, Kerry A Naish, Thomas P Quinn

**Affiliations:** School of Aquatic and Fishery Sciences, University of WashingtonSeattle, WA, USA

**Keywords:** admixture, artificial propagation, assignment test, hatchery management, hybridization, microsatellite DNA, *Oncorhynchus mykiss*

## Abstract

Two strategies have been proposed to avoid negative genetic effects of artificially propagated individuals on wild populations: (i) integration of wild and captive populations to minimize domestication selection and (ii) segregation of released individuals from the wild population to minimize interbreeding. We tested the efficacy of the strategy of segregation by divergent life history in a steelhead trout, *Oncorhynchus mykiss*, system, where hatchery fish were selected to spawn months earlier than the indigenous wild population. The proportion of wild ancestry smolts and adults declined by 10–20% over the three generations since the hatchery program began. Up to 80% of the naturally produced steelhead in any given year were hatchery/wild hybrids. Regression model selection analysis showed that the proportion of hatchery ancestry smolts was lower in years when stream discharge was high, suggesting a negative effect of flow on reproductive success of early-spawning hatchery fish. Furthermore, proportions of hybrid smolts and adults were higher in years when the number of naturally spawning hatchery-produced adults was higher. Divergent life history failed to prevent interbreeding when physical isolation was ineffective, an inadequacy that is likely to prevail in many other situations.

## Introduction

Goals associated with commercial and recreational use of wild animal populations often compete with conservation efforts. A common strategy to alleviate this competition is to release large numbers of artificially propagated individuals into the wild. Millions of insects, birds, trees, fish, and other animals have been released into natural habitats around the globe (Laikre et al. 2010). These releases are often intended to support declining populations, direct effort away from wild populations, mitigate habitat losses, and provide harvest opportunities (Taylor 1999; Waples 1999; Carroll 2011). The underlying philosophy is that activities such as fishing, hunting, and forestry might be sustainable if they are directed toward surplus individuals. However, the potential for negative ecological (Duncan et al. 2003) and genetic (Laikre et al. 2010) impacts of such releases on wild conspecifics is widely recognized, and methods of reducing or eliminating these impacts while allowing continued artificial propagation are increasingly important (Mobrand et al. 2005; Lankau et al. 2011).

Concerted efforts at reducing the impacts of releases on native populations are growing in fisheries management (Leber et al. 2004; Bell et al. 2005; Lorenzen et al. 2010). In salmonid fishes (*Oncorhynchus*, *Salmo*, *Salvelinus* spp.) in particular, captive rearing has occurred for over a century in part to offset losses from habitat degradation, overfishing, and climate-driven marine and freshwater changes (National Research Council 1996; Stouder et al. 1996). Hatchery-produced salmonids are routinely released to mingle and interact with their wild counterparts at some point in their lifetime, and considerable attention has been focused on defining and quantifying the benefits and risks associated with such practices (Naish et al. 2008; Kostow 2009; Araki and Schmid 2010). Reviews of the genetic impacts of hatcheries on wild populations (e.g., Hindar et al. 1991; Waples 1991; Busack and Currens 1995; Ryman et al. 1995; Naish et al. 2008) have identified three important processes: (i) effects of hatcheries on the fitness of hatchery fish, and (ii) direct and (iii) indirect effects of interactions between wild and hatchery populations. Many studies have debated the risk associated with these effects (e.g. Waples 1999; Brannon et al. 2004), but interest currently centers on the degree to which this risk is realized, and what management steps can be taken to minimize the impacts of hatchery fish on wild fish (Campton 1995; Waples 1999; Mobrand et al. 2005; Naish et al. 2008).

Recent analyses of hatchery practices have resulted in specific recommendations aimed at reducing the genetic risks associated with hatchery fish. Two main approaches have been suggested: hatchery populations should be either genetically integrated with, or segregated from, natural populations (e.g. Mobrand et al. 2005) by promoting or restricting gene flow. ‘Integration’ requires that each hatchery population be managed as a small, artificially propagated component of the local natural population, where broodstock (adults bred in captivity) is replenished frequently from the wild to minimize genetic divergence between the two populations. Alternatively, ‘segregated’ programs are designed to maintain genetically and ecologically discrete hatchery and wild populations that can be managed as separate entities, thus minimizing interactions between the two components.

Segregation of salmonid populations is typically achieved by marking hatchery-produced fish and using migration barriers such as dams, weirs, or traps to selectively allow only unmarked wild fish access to spawning grounds (e.g. Mclean et al. 2003; Araki et al. 2007a). Weirs and traps may not exist on all rivers where hatchery salmonids are released and even when they are in place they are imperfect barriers (Quinn 1993). Moreover, hatchery-produced fish may adopt a nonanadromous life history (Christie et al. 2011a), further facilitating interbreeding with wild fish. Notwithstanding these difficulties in segregating the populations, the use of propagated individuals selected for very different life history traits can enhance separation between hatchery and wild stocks (Lorenzen et al. 2010), but the efficacy of such measures has not been fully tested.

The winter-run (ocean maturing) anadromous steelhead (*Oncorhynchus mykiss*) hatchery programs of Washington State, USA, rely almost exclusively on the use of a single broodstock that has been artificially selected to have a life history pattern divergent from that of most wild winter-run steelhead populations (Crawford 1979; Mobrand et al. 2004). This stock, which originated from Chambers Creek in Puget Sound, Washington, returns to freshwater to spawn several months earlier than most wild populations in the Pacific Northwest (Chambers Creek, November–January; wild, February–May; Busby et al. 1996). This differential timing is thought to prevent interbreeding between hatchery-produced and wild fish, and to facilitate harvest of hatchery-produced fish with little to no impact on later-migrating wild fish. Ecological interactions might be further minimized because the hatchery fish spawn during the winter, when higher stream flows often scour the gravel nests, increasing mortality of developing embryos (Montgomery et al. 1999). The combination of temporally separate breeding and maladaptive timing is believed to minimize or eliminate most risks to wild populations associated with hatchery production using this approach.

A new steelhead hatchery program at Forks Creek Hatchery was initiated in 1994 with the release of Chambers Creek ancestry smolts (juveniles ready to migrate to sea). We have genetically monitored the steelhead in Forks Creek since the winter 1995–1996, the first year that the early-returning mature hatchery adults (the survivors of the smolts released in 1994) were spawned at Forks Creek Hatchery. Monitoring provided the opportunity to observe the effects of this hatchery program on the natural population from its inception. The specific aim of this study was to determine whether segregation based on differences in migration and spawn timing prevented hybridization of hatchery fish and wild fish and minimized ecological impacts by preventing natural propagation of hatchery fish. To achieve this aim, we evaluated temporal trends in the relative components of hatchery and wild steelhead in the naturally spawning population over the first three generations of the hatchery program. We then estimated the proportions of hatchery, wild, and hatchery/wild hybrid individuals in each annual collection using individual assignment data and evaluated possible factors influencing variation in these proportions.

## Methods

### Site description and sampling

Forks Creek is a small tributary to the Willapa River in southwest Washington State, USA (map in Fig. 1 of Mclean et al. 2004). The Washington Department of Fish and Wildlife (WDFW) operates a salmon and steelhead hatchery (Forks Creek Hatchery) located on Forks Creek upstream of its confluence with the Willapa River. Juvenile hatchery-produced fish are marked by removal of their adipose fin before release from the hatchery, thus they can be visually distinguished from naturally produced (unmarked) fish when they return as mature adults. We sampled steelhead at the Forks Creek Hatchery annually from the initiation of propagation there in fall 1995 through the winter of 2009–2010. Returning adults were diverted from the creek by a permanent weir into a shallow hatchery pond where they were seined and sampled. Marked fish were killed at the hatchery in all but the first 2 years of the project, when they were allowed upstream in Forks Creek. All unmarked adults were sampled and then placed back in Forks Creek above the weir. In principle, the weir diverted all migrating adults into the hatchery. However, observations indicated it was not fully effective at high stream flows, and many unsampled adults jumped over the weir and continued upstream. Therefore, pre- and postspawning adult steelhead were also sampled by tangle net and angling in the creek. Postspawning adult steelhead (kelts) and smolts migrating downstream were captured in a fan-type trap installed in the weir and operated from early April through early June. The return timing of a single cohort of adults commonly spans two calendar years (typically November-January for hatchery fish and February-April for wild fish), but for the purposes of analysis, they were labeled as a single return or brood year using the year starting in the wild population spawning season. Sampling date, sex (adults only), fork length, and body weight were recorded, and scales (for age determination, from adults only) and a small piece of caudal fin tissue for DNA analysis were obtained from all trapped or captured fish. Scales and fin tissue were immediately preserved in 95% ethanol.

**Figure 1:**
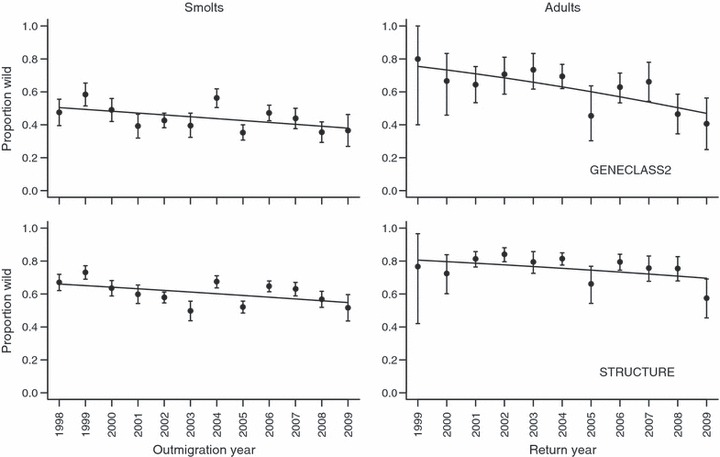
Time series of the mixture (geneclass2) and admixture (structure) proportions of samples of outmigrating smolt and adult steelhead genetically identified as having wild ancestry. Error bars represent the 95% confidence interval from wild mixture proportions or average admixture bootstrapped across individuals. Solid lines are the predicted values from GLM analysis.

### Genetic methods

Genomic DNA was extracted and isolated from fin tissue using Qiagen DNEasy tissue extraction kits (Qiagen Inc., Valencia, CA, USA). We genotyped all fish at eight microsatellite loci (Table S1) as described in detail in Mclean et al. (2004). Polymerase chain reaction products were size-fractionated and visualized on the MegaBACE 1000 capillary electrophoresis system (GE Healthcare, Piscataway, NJ, USA). Electropherograms were analyzed using Genetic Profiler (GE Healthcare).

### Dataset selection

Our analyses of the proportion of hatchery-origin fish produced in the wild relied on unmarked adult and smolt steelhead spawned in the river. Resident and anadromous coastal cutthroat trout (*Oncorhynchus clarki clarki*) are also found in Forks Creek, but this species, steelhead, and their hybrids can be difficult to distinguish visually from one another (Baumstieger et al. 2005). Therefore, we identified cutthroat and hybrids using NewHybrids (Anderson and Thompson 2002). NewHybrids performs a Bayesian analysis to determine the posterior probability that an individual belongs to one of 6 hybrid classes (pure steelhead, pure cutthroat, F1 hybrid, F2 hybrid, steelhead backcross, or cutthroat backcross). We used uninformative Jeffreys-like priors, which accomodate low-frequency alleles in both species. In the analyses, 674 adult hatchery-produced steelhead were included as baseline information for steelhead, 10 000 iterations for burn-in were used, followed by 100 000 iterations. Species class for each individual was designated as the class with the highest posterior probability. All fish identified as cutthroat or hybrids of any class were eliminated from further analyses.

We used all genotype data from Hauser et al. (2006), which comprised genotypes of the baseline populations and the first 3 years of adult returns, and added all smolt genotypes and adult genotypes from years 2002 to 2009. Parentage analysis previously revealed that many unmarked adult steelhead returning to Forks Creek in 1999–2001 were offspring of fish spawned in the hatchery (Hauser et al. 2006). These fish had either escaped the hatchery before they could be marked or marking was incomplete (Thompson and Blankenship 1997). We performed parentage analysis, as described by Hauser et al. (2006), on all subsequent adult collections as well as on smolt collections to identify and remove these fish from the analyses.

### Genetic assignment and admixture

Genetic mixture proportions in the naturally produced fish were estimated using two methods: (i) from results of individual assignment tests, which assume no admixture, and (ii) from results of admixture analysis. Individual assignment tests were carried out using geneclass2 (Piry et al. 2004) using Bayesian methods (fully described in Hauser et al. 2006). Individuals were assigned to either hatchery or wild ancestry by summing the probabilities of belonging to baseline collections. Individuals with a probability ≥0.95 were classified as hatchery or wild (‘confident assignment’). Those with probabilities <0.95 were given ‘unassigned’ ancestry. Wild mixture proportions were calculated as the number of wild ancestry individuals divided by the total sample (including unassigned individuals). Admixture proportions of genetic contribution to hybrid individuals were estimated using the program structure (Pritchard et al. 2000; Falush et al. 2003). All smolt and adult annual collections were run together in one single run with each collection labeled as a separate population. We ran 100 000 iterations as burn-in and another 100 000 for analysis with the number of populations set at *k* = 2 (Hauser et al. 2006). Convergence was assessed by inspection of alpha values per iteration. Wild ancestry proportions per collection were calculated as the average individual wild admixture (*q*). We used both individual assignment (geneclass2) and admixture analysis (structure) because geneclass2 was more accurate than structure in assigning individuals to hatchery or wild ancestry (Hauser et al. 2006), but in contrast to structure, the program did not permit interbreeding between groups. To simplify text, results from geneclass2 analysis are referred to as mixture proportions and those from structure are referred to as admixture proportions.

We included baseline collection genetic data on wild steelhead in analyses with both methods, consisting of the unmarked adults returning in brood years 1996, 1997, and 1998. Brood year 1999 was the first year that offspring of naturally spawning hatchery-produced steelhead could have returned to spawn, so samples from the three prior brood years were likely to be exclusively wild ancestry individuals. Our baseline hatchery collections consisted of marked (adipose fin clipped hatchery-produced) fish returning in the same years: 1996, 1997, and 1998. Using approaches fully described in Hauser et al. (2006), the quality of our baseline genetic data was evaluated by estimating deviations from Hardy–Weinberg equilibrium (HWE) using Genepop v3.4 (Rousset 2008). *F*_IS_ values and allelic diversity were calculated using Fstat (Goudet 1995). Significant deviations from HWE were found in baseline populations, in part due to a null allele in locus *Omy*77, but these were found to have little to no effect on assignment success (Hauser et al. 2006).

To maximize our sample size without adding uncertainty, we evaluated the effect of missing genotypes on individual assignment test results. We randomly removed genotypes of one locus per individual from original datasets comprising fish with complete genotypes and re-analyzed the data in geneclass2. This procedure was repeated until all but one locus had been deleted. The fractional change in unassigned individuals with each increase in missing loci was used to determine the minimal number of loci necessary for accurate assignment. Individuals with fewer genotypes than the minimum number were removed from further analysis.

### Time series trend analysis

Using the results of the assignments, we examined temporal trends in the proportions of wild ancestry smolts in years 1998 through 2009, and wild ancestry adults returning to spawn in years 1999 through 2009. Autocorrelation among annual collections within each dataset (smolt and adult) with a time lag of up to 5 years (maximum age at maturity of Forks Creek steelhead, Dauer et al. 2009) was tested by the Durbin–Watson statistic. The significance of *P*-values of these tests was corrected using false discovery rate (Verhoeven et al. 2005). If there was no evidence of autocorrelation, the data were evaluated using a beta regression (Simas and Rocha 2010) with proportion wild as the dependent variable and year as the independent variable. We used beta regression because the dependent variable was a proportion but not strictly binomial. Analysis was performed in r (package ‘betareg’, R Development Core Team 2009 2009; Cribari-Neto and Zeileis 2010), with a loglog link and beta distribution error structure. We repeated the smolt analysis without the first outmigration year (1998), because this collection likely included a small but unknown number of age 3 individuals spawned in 1995, before our study started. Similarly, we repeated the adult analysis without return years 1999 and 2000, because the first year of adult returns (1999) had a very small sample size (*n* = 5) and return years 1999 and 2000 included only partial cohorts spawned in 1996 and 1997. We performed a resampling analysis to propagate the uncertainty in the original mixture proportion estimates through to the analysis of temporal trends (Supporting information). Briefly, bootstrap distributions encompassing each mixture or admixture proportion estimate were resampled and the beta regression was performed 10 000 times. Support for the regression results of the time series analyses was gauged by the fraction of regressions with a significant slope (*P* < 0.05) and a slope of the same sign as that of observed data.

Mixture proportions calculated using individual assignment may be less accurate than those computed directly using fractional allocation methods (Manel et al. 2005), so we performed the same regression analysis using estimates calculated by a maximum likelihood estimator (oncor, Anderson et al. 2008) and a Bayesian estimator (bayes, Pella and Masuda 2001).

### Estimating true hatchery, wild, and hybrid mixture proportions

Interbreeding may have occurred between hatchery-produced or hatchery ancestry (naturally produced) and wild steelhead. Our genetic dataset lacked the statistical power to explicitly estimate hybrid classes of individuals (e.g. via NewHybrid). However, using observed mixture proportions (hatchery, wild, unassigned) and estimated assignment rates of our test (geneclass2), we could estimate the true proportions of hatchery, wild, and hybrid individuals in our annual collections. Assignment rates of pure hatchery, pure wild, and hybrid individuals were estimated by simulating 100 000 each of pure hatchery, pure wild, and F1 hatchery/wild hybrid genotypes with hybridlab (Nielsen et al. 2006) and our baseline genetic data. Simulated data were analyzed using geneclass2 using confident assignment, and assignment rates for each group (hatchery, wild, and unassigned) were calculated. Those rates were then used to compile three equations, one for each class of individual assignment with confident assignment, with three unknowns, the true proportions of hatchery, wild, and hybrid individuals.


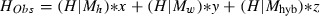
(1)


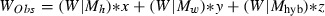
(2)


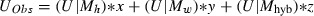
(3_

*H*, *W*, and *U* are observed (Obs) hatchery, wild, and unassigned mixture proportions (respectively) estimated by geneclass2 assignment; *M_h_*, *M_w_*, or *M_hyb_* are hatchery, wild, and unassigned mixture proportions of simulated hatchery, wild, or hybrid genotypes; and *x*, *y*, and *z* are the unknown true hatchery, wild, or hybrid mixture proportions. True proportions were estimated by solving this system of equations for each annual smolt and adult collection using linear algebraic methods in r. Uncertainty in the true proportions was estimated by resampling individual assignments (with confident assignment), calculating the proportions of each assignment class, and solving for unknown true proportions. Resampling was performed 10 000 times. Reliable confident assignment of individuals estimated with structure could not be obtained because of limited power of the microsatellite marker set and because individual admixture estimates varied with mixture proportions in the simulated dataset (data not shown), so this analysis was performed using only geneclass2 individual assignments.

### Evaluating explanatory variables (model selection)

We used forward stepwise model selection and information theoretic analysis to explore the effects of three factors that might have affected the patterns in mixture proportions: (i) the number of hatchery-produced adult steelhead spawning in Forks Creek upstream of the hatchery, (ii) the number of wild fish in the river, and (iii) river discharge during the period when hatchery steelhead were spawning. The numbers of hatchery and wild fish are important because the numbers of pure hatchery and wild offspring in a sample should be proportional to the parental numbers and because the opportunity for interbreeding may also be influenced by numbers of fish. River discharge could be important if floods early in the winter allowed migration over physical barriers (to the benefit of hatchery-produced fish) or if they reduced the reproductive success of naturally spawning hatchery-produced or hatchery ancestry fish relative to wild fish spawning later, when flows are more moderate (Mackey et al. 2001).

We used the annual total number of hatchery-produced adults returning to the hatchery as an index of the number of hatchery-produced fish spawning upstream of the weir, and we obtained independent estimates of the number of wild fish spawning in the entire Willapa River basin made by state fisheries biologists (WDFW 2011). These estimates were based on counts of redds (nests dug by female steelhead) in index reaches of the Willapa River and tributaries from 16 March through May of each year. We used each of these indices individually and together as the hatchery-produced adult proportion index [hatchery index/(wild index + hatchery index)] as covariates in model selection analysis. Because each index was estimated in different ways and for different spatial scales, the proportion is unit-less. Because the proportion of hybrids should increase only as the proportion of wild (or hatchery) spawners approaches 0.5 (a quadratic relationship), we analyzed one model for proportion of hybrids for each life history that included the hatchery-produced adult proportion index and a squared hatchery-produced adult proportion index. Discharge data were obtained from the United States Geological Survey (USGS) monitoring station on the Willapa River, 30 km downstream of Forks Creek (USGS 2011). We calculated the average of average daily discharge data from December through February of a brood year (e.g. December 1995 through February 1996), which encompasses the spawning timing of the majority of hatchery-produced fish. We also included year as a covariate. Models were tested using beta regression analysis in r with a loglog link and beta distribution error structure. Relative support for these variables was evaluated based on corrected Akaike information theoretic values (AIC_*c*_, Burnham and Anderson 2002). Estimated true mixture proportions were lagged 2 years for smolt samples and 4 years for adult samples because the most common ages were 2 years (age at outmigration) for smolts and 4 years (total age) for adults (Busby et al. 1996; Dauer et al. 2009). Cohort analysis was not possible because of limited age data for smolts and unmarked adults.

### Genetic drift

Large and unequal rates of genetic drift between hatchery and wild populations since the initiation of the study may have affected individual ancestry assignments and could influence trends in the time series. The rate of genetic drift is directly proportional to the effective population size (*N*_*e*_), so we estimated the *N*_*e*_ of the wild and hatchery adult baseline collections to assess the potential influence of genetic drift. We calculated *N*_*e*_ from only the baseline samples because later collections were an unknown mix of hatchery and wild samples. To estimate *N*_*e*_, we used the single sample linkage disequilibrium methods implemented in the program ldne (Waples and Do 2008). As estimates of *N*_*e*_ from linkage refer to previous generations, genotypes from all baseline hatchery fish collections (released or killed) and wild fish collections were combined into two groups consisting of either hatchery or wild individuals. We used a *P*_crit_ value (the minimum frequency at which alleles were included in the analysis) of ≥0.05 (Waples and Do 2010) as the cutoff point in our analyses.

### Analysis of life history traits

Migration timing of the hatchery and wild steelhead populations was considered a key mechanism to ensure segregation. To evaluate whether adult migration timing was maintained under natural spawning and rearing conditions, differences in the upstream migration date of hatchery, wild, and unassigned individuals were tested using anova. Migration date in salmonids can vary with return year, sex, and fish length (Quinn 2005), so these variables and factors were included with ancestry as factors in the analysis.

Among smolts, we tested for differences in fork length, body weight, age, and date that each fish passed the weir, and among adults, we tested for differences in fork length and age between hatchery and wild ancestry individuals. Outmigration or return year and ancestry were included as factors in all analyses. Sex was included as an additional factor in all adult analyses, and age was included as an additional factor in analysis of adult fork length. Adults identified as repeat spawners were omitted from analysis of length and age. Smolt age at outmigration was estimated from parentage data for 281 smolts (see Hauser et al. 2006 for parentage methods), and differences between hatchery and wild fish were tested by χ^2^ contingency table.

## Results

A total of 4645 smolts and 1447 returning adults were sampled between 1996 and 2009. The baseline populations included 674 adults returning the first 3 years; 563 of these were hatchery-produced fish (see Table 2 in Hauser et al. 2006). We identified 621 smolts and seven adults as possible cutthroat trout or cutthroat/steelhead hybrids and excluded them from further analysis. We found 38 pairs of adult samples (and no smolts) that shared identical genotypes at all eight loci; these fish were either individuals that were sampled twice during their upstream and downstream migrations or fish that were sampled in consecutive years (‘repeat spawners’). Each of these individuals was counted only once for further analysis; repeat spawners were included in the return year of their first spawning migration. Individual assignment success using geneclass2 changed very little when five or more loci were used in the assignment tests (Fig. S1); therefore, all fish genotyped at five or more loci were used in further analyses. Our final test dataset included 3107 smolt samples and 657 adult samples (Table 1).

**Table 1:** Sample sizes of data on steelhead used in analyses by year sampled

	Final sample size	
	
Year sampled*	Smolts	Adults
1998	162	–
1999	202	5
2000	200	24
2001	166	73
2002	476	58
2003	170	60
2004	309	150
2005	420	33
2006	424	105
2007	260	59
2008	225	58
2009	93	32
Total	3107	657

* Year sampled was, for smolts, outmigration year, and for adults, the year of their spawning migration.

**Table 2:** Rates of individual assignment (geneclass2) of 100 000 genotypes each of pure hatchery, F1 hybrid, and pure wild simulated from baseline collection genotypes

		True ancestry (simulations)		
		
		Hatchery (*h*)	F1 Hybrid (hyb)	Wild (*w*)
Estimated ancestry (GENECLASS2)	Hatchery (*H*)	0.943	0.373	0.007
	Unassigned (*U*)	0.056	0.411	0.126
	Wild (*W*)	0.001	0.216	0.867

*F*_ST_ estimates among collections and cohorts varied between 0.002 and 0.036, and the average *F*_ST_ among baseline collections was 0.015 (Table 3 in Hauser et al. 2006). The estimate of *N*_*e*_ for the parents of the wild baseline was 204 (95% CI = 141–338), and for the parents of the hatchery baseline, it was 226 (153–353).

### Time series trend analysis

No significant autocorrelation was found at any time lag in either the smolt or the adult dataset (*P* ≫ 0.05, five tests each for smolts and adults), so we proceeded with the time series analysis. The proportion of wild ancestry fish declined over time in both smolt and adult datasets using either mixture (geneclass2) or admixture (structure) proportions (Fig. 1; *P* < 0.05 all analyses, Table S2). The negative trend was well supported by the data; 92–100% of the resampled datasets had negative slopes (Table S2), but only about half of the tests of smolt and adult mixture and admixture datasets were statistically significant (*P* < 0.05; Table S2). Re-analysis excluding the early years of smolt and adult datasets produced slightly different parameter estimates and had little effect on the trends in the sign of the slope. However, the number of statistically significant regressions of smolt data decreased, and the number of statistically significant regressions of adult data increased (Table S3). Results using mixture proportion estimates from oncor and bayes were similar to those using geneclass2 and structure (Tables S2 and S3), so all further analyses were conducted only with mixture or admixture proportions estimated by geneclass2 and structure.

### Estimated true mixture proportions

Simulated pure hatchery and wild genotypes were correctly assigned back to hatchery and wild groups at high rates and incorrectly assigned to the opposite group at very low rates by geneclass2 (Table 2). Simulated F1 hybrid genotypes were assigned back to hatchery, wild, and unassigned groups at roughly equal rates, with the highest proportion being categorized as unassigned (Table 2). Single solutions were found for all annual collections (Table 3). However, estimates for two smolt collections and five adult collections included negative hatchery proportions that were not biologically meaningful (Table 3). Further analysis proceeded without these hatchery proportions.

**Table 3:** Estimates of the true proportions of hatchery, wild, and hatchery/wild F1 hybrid individuals in Forks Creek

	Smolts			Adults		
		
Sample year	Hatchery	F1 Hybrid	Wild	Hatchery	F1 Hybrid	Wild
1998	−0.066	0.690	0.376	–	–	–
1999	0.026	0.401	0.573	–	–	–
2000	−0.043	0.637	0.406	0.103	0.172	0.725
2001	0.020	0.704	0.276	−0.173	0.574	0.599
2002	0.149	0.480	0.371	−0.025	0.280	0.745
2003	0.269	0.369	0.362	0.003	0.203	0.794
2004	0.022	0.439	0.539	−0.041	0.323	0.718
2005	0.237	0.477	0.286	0.007	0.624	0.369
2006	0.051	0.540	0.409	−0.060	0.447	0.613
2007	0.021	0.631	0.348	0.028	0.280	0.692
2008	0.017	0.763	0.220	−0.173	0.846	0.327
2009	0.145	0.577	0.278	0.104	0.570	0.326

### Evaluation of explanatory variables

**Figure 2:**
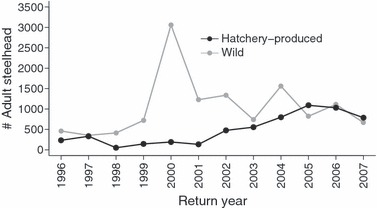
The number of hatchery-produced (marked) adult steelhead (black) returning to Forks Creek Hatchery and the estimated number of wild adult steelhead (gray; WDFW 2011) returning to the Willapa River from 1996 through 2007.

**Figure 3:**
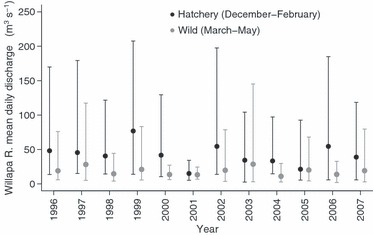
Average daily discharge from the Willapa River (m^3^ s^−1^) during hatchery (December through February, in black) and wild (March through May, in gray) steelhead migration and spawning from brood years 1996 through 2007. Bars represent 0.95 quantiles.

The number of hatchery-produced adult steelhead returning to Forks Creek Hatchery ranged from 51 in 1998 to 1091 in 2005 (Fig. 2). Over the same period, the number of wild adult steelhead estimated in the entire Willapa River basin ranged from 355 adults (1997) to 3059 adults (2000) (Fig. 2). This resulted in hatchery-produced adult proportion indices that ranged from 0.11 (1998) to 0.54 (2007), a range consistent with previous estimates of the proportion of naturally spawning hatchery-produced adults (Dauer et al. 2009). The overall average daily discharge of the Willapa River during hatchery steelhead migration and spawning (December through February) of brood years 1996 through 2007 was high (42.1 m^3^ s^−1^), and annual average daily discharge varied fivefold among years (15.2–76.9 m^3^ s^−1^, Fig. 3). The overall average daily discharge during wild migration and spawning (March through May) was less than half that of the hatchery spawning months (19.0 m^3^ s^−1^) and varied annually less than threefold (11.0–28.6 m^3^ s^−1^, Fig. 3). The number of hatchery-produced fish returning to the hatchery increased over time (Fig. 2), but collinearity was below the threshold where it is typically considered a problem for multiple regression (VIF < 5; O’Brien 2007). No other predictor variables were correlated with any others.

**Figure 4:**
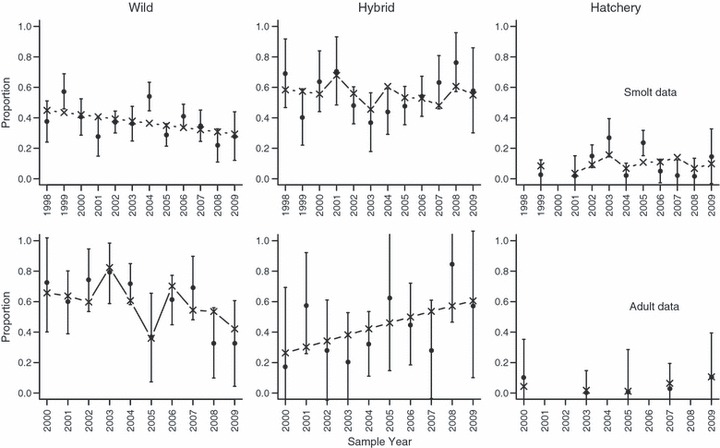
Time series of estimates of true proportions of wild, hatchery, and hatchery/wild hybrid smolt and adult individuals (black) and expected values from the best fit models (-**×**-; Table S4). Error bars represent 95% confidence interval of true estimates based on distributions of observed hatchery and wild mixture proportions bootstrapped across individuals.

Estimated proportions of wild smolts and adults declined over time at approximately the same rate that hybrid proportions increased, and hatchery proportions changed very little with time (Fig. 4, Table S4). There was no strong support for any one model over most other models evaluated with smolt data (ΔAIC_*c*_ < 10, Tables 4 and S4) (Burnham and Anderson 2002). The model predicting wild smolt proportions and including year as the only covariate had the smallest AIC_*c*_ (i.e. best fit model). For models predicting hybrid and hatchery proportions, the smallest AIC_*c*_ were found when the models included winter stream discharge as the only covariate; increased discharge corresponded to higher proportions of hybrid smolts and lower proportions of hatchery ancestry smolts. Coefficients for the single covariates in these three best fit models were not statistically significant after correction for multiple tests using false discovery rate. The model predicting wild adult proportions and including winter stream discharge as the only covariate had the smallest AIC_*c*_ (Fig. 4, Tables 4 and S4). The best fit model for predicting hybrid proportions included year as the only covariate. The best fit model predicting hatchery proportions included the hatchery-produced adult proportion index as the only covariate (Fig. 4, Tables 4 and S4). No one model was more plausible than the others (ΔAIC_*c*_ < 10, Tables 4 and S4). Coefficients for covariates in the best fit models predicting hatchery and wild proportions were statistically significant after correction for multiple tests by false discovery rate. Models run using the hatchery-produced adult proportion index generally had similar AIC_*c*_ values as models run using the hatchery-produced adult number index for both smolt and adult datasets. There was no obvious quadratic relationship of the hatchery-produced adult proportion index with hybrid smolt or adult proportions.

**Table 4:** The five models with the smallest AIC_*c*_ for each life history and class from results of model selection analysis

Life history	Class	Model	Model rank	AIC_*c*_	ΔAIC_*c*_	Coeff. 1	*P*-value 1	Coeff. 2	*P*-value 2	Coeff. 3	*P*-value 3
Smolt	Wild	Sampyear	1	−16.29	0.0	−0.03855	*0.04720*				
		Hatchnum	2	−14.24	2.1	−0.00025	0.22600				
		Hatchprop	3	−13.13	3.2	−0.22680	0.60200				
		Discharge	4	−12.95	3.3	−0.00147	0.76500				
		Wildnum	5	−12.86	3.4	0.00000	0.99700				
	Hybrid	Discharge	1	−10.81	0.0	0.01144	0.07000				
		Hatchnum	2	−8.78	2.0	0.00029	0.32950				
		Discharge, hatchnum	3	−8.72	2.1	0.01324	**0.02050**	0.00041	0.10200		
		Wildnum	4	−8.68	2.1	−0.00014	0.32272				
		Hatchprop	5	−8.32	2.5	0.44300	0.46030				
	Hatchery	Discharge	1	−19.64	0.0	−0.00947	0.08180				
		Discharge, hatchnum	2	−18.18	1.5	−0.01429	**0.00290**	−0.00057	**0.01420**		
		Hatchprop	3	−17.85	1.8	−0.55210	0.32802				
		Hatchnum	4	−17.86	1.8	−0.00026	0.36328				
		Discharge, hatchnum, sampyear	5	−17.76	1.9	−0.01349	**0.00012**	−0.00148	**0.00000**	0.12415	**0.00033**
Adult	Wild	Discharge	1	−6.53	0.0	0.02695	**0.00034**				
		Discharge, hatchnum	2	−5.12	1.4	0.02194	**0.00088**	−0.000672	**0.01580**		
		Hatchnum	3	−4.33	2.2	−0.00112	**0.00217**				
		Sampyear	4	−4.28	2.3	−0.13195	**0.00320**				
		Discharge, sampyear	5	−3.85	2.7	0.01962	**0.00731**	−0.07824	*0.04602*		
	Hybrid	Sampyear	1	1.81	0.0	0.10856	*0.03830*				
		Hatchnum	2	1.97	0.2	0.00104	*0.04870*				
		Discharge	3	2.14	0.3	−0.01778	*0.04520*				
		Hatchprop	4	4.76	2.9	−0.38132	0.39200				
		Wildnum	5	5.07	3.3	0.00016	0.49200				
	Hatchery	Hatchprop	1	4.72	0.0	1.44270	**0.00537**				
		Hatchnum	2	6.45	1.7	0.00058	**0.03320**				
		Wildnum	3	8.47	3.8	−0.00050	0.26500				
		Discharge	4	8.95	4.2	−0.00345	0.51618				
		Sampyear	5	9.35	4.6	0.01940	0.61300				

Sampyear, sample year; hatchnum, index of hatchery adults; wildnum, index of wild adults, hatchprop, hatchery-produced adult proportion index; discharge, stream discharge during hatchery fish migration and spawning. The full table is available in the Supporting information Table S4.

*P*-values in boldface were significant after correcting for multiple tests using false discovery rate. *P*-values in italics were significant before correcting for multiple tests.

### Life history trait patterns

**Figure 5:**
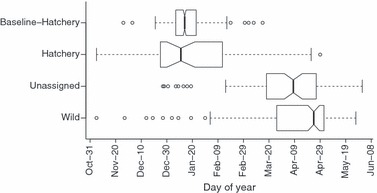
Box and whisker plot of the spawning migration timing of baseline hatchery adult steelhead and of adult steelhead assigned to hatchery, unassigned, and wild ancestry using geneclass2 with confident assignment criteria. No upstream migration timing data from wild baseline samples was available.

Adult hatchery ancestry steelhead migrated early, with annual median migration dates between 27 December and 3 March and an overall median migration date of 12 January (Fig. 5). Migration timing of wild and unassigned ancestry adult steelhead was much later than hatchery ancestry fish (*F*_(2,216)_ = 47.56, *P* << 0.001) with annual median migration dates between 23 March and 29 May and an overall median of 26 April. Median annual upstream migration dates for baseline hatchery adults were between 10 and 24 January. Despite the differences in medians, there was considerable overlap in migration dates among all groups. We could not calculate a median upstream migration date for baseline wild adults because they were all sampled as they exited Forks Creek after spawning.

Smolt length, weight, and migration date varied among years (*F*_(11,3088)_ = 16.96, *P* << 0.001, length; *F*_(11,2936)_ = 11.24, *P* << 0.001, weight; *F*_(11,3903)_ = 36.88, *P* << 0.001, migration date), but trait values did not differ among hatchery, unassigned, and wild ancestry groups (*F*_(2,3088)_ = 0.54, *P* = 0.58, length; *F*_(2,2936)_ = 0.73, *P* = 0.48, weight; *F*_(2,3903)_ = 0.88, *P* = 0.42, migration date). Hatchery ancestry smolts showed a younger age distribution (i.e. more age 1) than wild ancestry smolts (χ^2^_(0.05,4)_ = 17.36, *P* = 0.002, Table S5). Hatchery ancestry adults also tended to be younger than unassigned or wild ancestry fish (*F*_(2,201)_ = 2.86, *P* = 0.06), largely because there were more young males with hatchery ancestry. No significant relationship was detected between ancestry and returning adult fork length (*F*_(2,178)_ = 0.91, *P* = 0.403).

## Discussion

Our aim was to evaluate whether segregation by life history was an effective management strategy for minimizing or eliminating genetic interactions between wild and hatchery populations. Despite the earlier spawn timing in the hatchery population, our data suggest that hatchery and wild steelhead interbred and produced ‘hybrid’ offspring. Interbreeding between hatchery and wild salmonids is not uncommon (e.g. Largiadér and Scholl 1996; Hansen et al. 2000; Araki et al. 2007b), but in this case, intentional selection for early return and spawn timing and use of a weir were thought to segregate the hatchery fish from wild conspecifics. Using estimates of mixture and admixture proportions, we found that the wild proportion of the annual number of outmigrating smolt and returning adult steelhead declined by 10–20% between 1998 (the first year offspring of hatchery fish would be detectable) and 2009 (our last year of sampling), or within about three generations. Estimates of the true proportions of wild adult and smolt steelhead also declined over time because of a reciprocal increase in the proportion of hybrid individuals. Although it was assumed that the weir spanning Forks Creek at the hatchery prevented upstream migration of most if not all adult steelhead (Mclean et al. 2003, 2004), we discovered that marked (hatchery-produced) steelhead spawned in the wild every year (Dauer et al. 2009), likely bypassing the weir during the moderate to high stream flows that are common in winter and spring. Thus, data suggest that a continual input of hatchery-produced fish resulted in a proportional increase in hatchery/wild hybrid individuals and a related decline in proportions of wild ancestry in the naturally spawning population.

Hatchery-produced adult steelhead typically migrated and spawned when the weir was least efficient at blocking their migration, potentially causing higher sampling efficiency of later-returning wild adults. This sampling bias may explain the higher proportions of wild fish observed in adults compared with smolts (Fig. 1). However, assuming that the bias did not change over time, it does not affect temporal trends in mixture proportions in either adults or smolts. It does, however, preclude direct comparison of mixture proportions between smolts and adults.

The methods used to calculate mixture and admixture proportions produced some erroneous assignments, which should not have affected our analysis. Assignment success with geneclass2 and structure was generally quite high when compared with parentage data (>90% success, Hauser et al. 2006), and simulations performed here also showed similarly high assignment success and low rates of assignment error. Genetic drift, which may have caused assignment error to change over time especially in the latest sample years, was unlikely to be a significant cause of assignment errors. Estimates of *N*_*e*_ from baseline cohorts of hatchery and wild steelhead were nearly equal and, assuming there was no significant decline in *N*_*e*_ since 1995 in either population, both were large enough that very little drift would be expected in the time span of our study (Waples 1989). The *N*_*e*_ of the hatchery-produced population, a likely contributor of offspring to our observed data, was also relatively large for each of four generations since the hatchery program began (*N*_*e*_ = 100; unpublished data).

Given the assignment rates of genotypes simulated from baseline genetic data, there were clearly more ‘unassigned’ individuals in the observed data than could be explained by mis-assignment of pure hatchery or wild individuals. The major assumptions of geneclass2 were that no admixed individuals were present in the observed or baseline data and that all baseline populations were sampled. Violation of either of these assumptions could have caused a large proportion of unassigned individuals. Steelhead exhibit high rates of natal philopatry (Shapovalov and Taft 1954; Quinn 2005), so it is unlikely that the baseline contained a high proportion of immigrants from unsampled populations. Any such strays would probably have been from nearby populations, which would likely be genetically similar to Forks Creek steelhead (Heggenes et al. 2006). The presence of a resident population of *O. mykiss* (rainbow trout) could similarly produce unassigned individuals. However, we have never captured a rainbow trout in Forks Creek despite extensive, multiyear electrofishing surveys. Snorkel surveys conducted in the Willapa River by Washington Department of Fish and Wildlife biologists likewise failed to detect any rainbow trout (J. Schultz, WDFW, unpublished data). Thus, the presence of hybrid individuals is the most plausible explanation for individuals unassigned to either wild or hatchery population.

Model selection analysis of factors that may explain observed variation in the proportions of wild, hybrid, and hatchery smolt and adult steelhead was inconclusive in that no one model explained the data better than the others. Although the model selection results should be considered exploratory, some interesting patterns were evident. As expected, winter stream discharge appeared to be an important factor explaining variation in the proportion of hatchery smolts. Higher stream discharge during hatchery spawning corresponded to smaller hatchery smolt proportions, which would be expected if high winter stream flows decreased the reproductive success of early-spawning hatchery ancestry steelhead. Mackey et al. (2001) noted that hatchery fish may be more successful in some winters with favorable spawning and juvenile rearing conditions. In 2001, the Willapa River’s condition during hatchery migration and spawning was similar in average and variance of discharge to that typical of conditions during migration and spawning of wild fish. These benign conditions probably explain the higher proportions of hatchery smolts in 2002 and 2003. However, higher proportions of hatchery fish were documented in 2005 and 2009 without correspondingly favorable flows in 2003 and 2007, suggesting that other mechanisms also determine reproductive success of hatchery fish. One explanation might be favorable conditions for hatchery ancestry fish during juvenile emergence rather than during spawning by their parents. Offspring of earlier spawning hatchery steelhead may emerge from the gravel (Mackey et al. 2001) and obtain feeding territories before wild ancestry fish. Earlier emerging fish will be larger (because they had time to grow) when the offspring of later-spawning fish emerge (Einum and Fleming 2000), and larger size provides fitness advantages (Abbott et al. 1985; Rhodes and Quinn 1998). A higher proportion of hatchery ancestry smolts migrated at age 1 rather than the typical age 2, consistent with these processes, because the age at which salmonids migrate to sea is inversely related to size or growth rate (Quinn 2005).

Winter stream discharge also corresponded to higher proportions of hybrid smolts. Higher winter discharge may have allowed a higher fraction of early-returning hatchery-produced adults upstream of the weir, increasing their opportunity to spawn with wild fish. It could also be an indirect result of the effect of discharge on early-spawning hatchery-produced adult reproductive success. That is, as a proportion, a direct reduction in hatchery ancestry fish must mean an increase in hybrid or wild (or both) proportions.

The number of returning hatchery adults in the parental generations (2 years before smolt outmigration, 4 years before offspring return) may also affect the of proportions of hatchery and hybrid origin smolts; though, the relationship was weak (very small regression coefficients). Using hatchery-produced adult proportion indices instead of the number of hatchery-produced adults in models did not significantly improve the models possibly because of the differing spatial scales of the wild and hatchery adult indices. The positive relationship of numbers of hatchery-produced adults on the spawning grounds with proportions of hybrid smolts was presumably because of increased opportunities for interbreeding on the spawning grounds as the number of hatchery fish on the spawning grounds increased. The lack of an obvious quadratic relationship of the hatchery-produced adult proportion index and hybrid smolts or adults was likely because the estimated proportion of hatchery-produced adults barely exceeded 0.50. Cohorts with more hatchery-produced parents also appeared to produce higher proportions of returning adult offspring of hatchery descent and lower proportions of wild ancestry smolts and adults. Counterintuitively, the relationship between the number of hatchery-produced adults on the spawning grounds and hatchery smolt proportions was consistently negative, suggesting that more hatchery-produced adults on the spawning grounds produced fewer hatchery descent smolts. The most likely reason for this somewhat unexpected relationship is the younger average age at seaward migration of hatchery ancestry smolts. That is, some smolts may have left Forks Creek already after 1 year and so were not included in the smolt count 2 years after the return of the parental generation. Unfortunately, not enough smolt ages were available to test this hypothesis.

The early migration and spawn timing of hatchery fish were believed to effectively prevent them from interbreeding with wild fish. Instead, it appears that significant proportions of the smolts and adults have been hatchery/wild hybrids. Earlier radio-tagging studies showed that there was some temporal overlap in migration and spawning, mainly hatchery-produced males arriving and spawning when wild fish were present (Mackey et al. 2001). There is considerable variation in migration and spawning timing even among wild fish in this and other steelhead populations (Seamons et al. 2007). Ample evidence shows that much of the variation in migration and spawn timing in salmonid fishes is attributed to additive genetic variation (Carlson and Seamons 2008). Thus, migration and spawn timing of hybrids would be expected to overlap with the timing of both populations. We were unable to explicitly identify hybrid individuals, but data showed that many individuals (especially the unassigned) were likely hybrids, and much of the overlap in migration timing seen in Fig. 5 might be due to these individuals.

Unassigned fish tended to spawn later than expected and closer to the spawning season of wild fish. This skew toward wild migration timing of putative hybrids may have several causes. First, it may be a result of selection against early migration. The decrease in hatchery ancestry proportions with increasing stream discharge during hatchery spawning provided evidence that early spawn timing is maladaptive, and genetic adaptation can occur in few generations (Christie et al. 2011b). Second, late-returning hatchery fish, especially males, may stay for several months in fresh water until wild females arrive (Leider et al. 1984; Seamons et al. 2004) and therefore may mate with wild fish throughout the wild spawning season, thus producing offspring with relatively late return timing. Third, genotype by environment interactions may cause a skew despite additive genetic variation of a trait. Little is known about the genetic architecture of return timing in salmonids, so this question needs further research. Finally, the apparent skew may be an artifact of differences in sampling efficiency at the weir during different stream flow regimes. With the exception of sampling artifacts, all these possible causes would lead to more substantial changes in migration timing of the naturally spawning population in the long run with concomitant negative fitness (Ford 2002). Although spawn timing may provide some reproductive isolation between fish of hatchery and wild descent (e.g. Hansen et al. 2006), a preponderance of hybrids spawning later and with larger temporal overlap with wild fish may accelerate the rate of introgression in the long term.

Controlling the behavior or breeding biology of captively reared animals released into the wild is one of the most significant issues for managers tasked with minimizing risks associated with captive rearing. The differential migration timing of hatchery and wild steelhead permits high harvest rates on the hatchery fish, followed by fishery closures during the later migrations of wild steelhead. However, differential harvest is an unreliable segregating mechanism because too many fish escape the fishery. Hatchery steelhead are intercepted and harvested downstream of the Forks Creek Hatchery, but harvest rates are clearly not sufficient to prevent large numbers of hatchery-produced fish from reaching spawning grounds (see also Dauer et al. 2009). Indeed, the number of hatchery-produced adults returning to the Forks Creek Hatchery equaled or exceeded the total number of wild fish estimated to be spawning in the entire Willapa River during the most recent three return years. Hatchery rearing may have negative fitness consequences even when the stocks are locally derived (Araki et al. 2007b, 2009). Nonlocal populations, like the hatchery broodstock used at Forks Creek, often have lower reproductive success than native wild populations because of a lack of local adaptation (Kostow et al. 2003; reviewed in Berejikian and Ford 2004; Araki et al. 2007a, 2008; Chilcote et al. 2011; Fraser et al. 2011). Interbreeding between hatchery and wild stocks could have long-term fitness consequences. One obvious solution is to reduce or cease production and release of steelhead from the hatchery; however, this option may be unpopular and difficult to implement. Physical segregation may be augmented by improving weirs. However, weirs or dams are costly and they affect the habitat to some extent. Flooding and debris compromise most weirs, allowing fish to bypass them. Even if barriers were completely effective at preventing upstream migration, the hatchery-produced fish might spawn elsewhere in the basin (Quinn 1993; Dittman et al. 2010). Segregation by life history was thought to complement physical segregation, but our study shows that it failed to prevent genetic interactions between hatchery and wild steelhead populations. Thus, managers should also consider other options for minimizing interactions between wild and cultured animals.

## Data archiving statement

Data deposited in the Dryad repository: doi: 10.5061/dryad.1cd96t8p.

## Supporting Information

**Figure S1.** The effect of missing genetic data on GENECLASS2 individual assignments.

**Table S1.** Microsatellite loci used forassignment testing, references for each locus, annealing temperature (T_A_), magnesium chloride concentration in mM and repeat unit in base pairs (bp).

**Table S2.** Parameter estimates from logistic regression analyses of time series data (*P*-values in parentheses) and resampling analysis.

**Table S3.** Parameter estimates from logistic regression analyses of time series data (*P*-values in parentheses) and resampling analysis excluding early data forsmolts (sample year 1998) and adults (sample years 1999, 2000).

**Table S4.** Results of model selection analysis.

**Table S5.** Proportions of smolts of wild, unassigned and hatchery ancestry outmigrating at 1, 2, or 3 years of age.

**Data S1.** Bootstrapping of beta regression analysis.
