# Supplementary material for: Can interbreeding of wild and artificially propagated animals be prevented by using broodstock selected for a divergent life history?
Source: Evol Appl. 2012 Nov;5(7):705–19. doi: 10.1111/j.1752-4571.2012.00247.x (PMC3492896; doi:10.1111/j.1752-4571.2012.00247.x)
Supplement: Supplementary file 1 [file eva0005-0705-SD1.pdf]

Supporting Information

Table S1. Microsatellite loci used for assignment testing, references for each locus, annealing temperature (T_A), magnesium chloride concentration in mM and repeat unit in base pairs (bp).

Locus	Source	T_A	[$MgCl_2$]	Repeat Unit (bp)
Oki23	A. Spidle[a]	55	1	4
Omy1001UW	Spies et al., 2005	55	1	4
Omy1011UW	Spies et al., 2005	55	1	4
Omy1191UW	Spies et al., 2005	65	1	4
Omy1212UW	Spies et al., 2005	65	1	4
Omy77	Morris et al., 1996	55	1	2
One108	Olsen et al., 2000	55	1	4
Ssa85	O'Reilly et al., 1996	60	2	2

[a] – Unpublished, GenBank accession # AF272822

Morris D. B., K. R. Richard, J. M. Wright. 1996. Microsatellites from rainbow trout (*Oncorhynchus mykiss*) and their use for genetic study of salmonids. Canadian Journal of Fisheries and Aquatic Sciences **53**, 120-126.

Olsen J. B., S. L. Wilson, E. J. Kretschmer, K. C. Jones, J. E. Seeb. 2000. Characterization of 14 tetranucleotide microsatellite loci derived from sockeye salmon. Molecular Ecology **9**, 2185-2187.

O'Reilly P. T., L. C. Hamilton, S. K. McConnell, J. M. Wright. 1996. Rapid analysis of genetic variation in Atlantic salmon (*Salmo salar*) by PCR multiplexing of dinucleotide and tetranucleotide microsatellites. Canadian Journal of Fisheries and Aquatic Sciences **53**, 2292-2298.

Spies I. B., J. D. Brasier, P. T. O'Reilly, T. R, Seamons, P. Bentzen. 2005. Development and characterization of novel tetra-, tri-, and dinucleotide microsatellite markers in rainbow trout (*Oncorhynchus mykiss*). Molecular Ecology Notes **5**, 278-281.

Bootstrapping of logistic regression analysis

We performed a resampling analysis to propagate the uncertainty in the original mixture proportion estimates through to the analysis of temporal trends. First, we performed individual assignment tests with ‘confident assignment’ criteria. We then defined the distributions of uncertainty for each individual assignment approaches, since these distributions differ between the approaches. Distributions of uncertainty were estimated by first calculating bootstrap 95% confidence intervals for each annual collection by sampling 10,000 times with replacement from the individual assignment data, then calculating the proportion wild for each of the 10,000

samples for each annual collection. Admixture proportions estimated by STRUCTURE were within-sample average individual admixture proportions. Uncertainty in admixture estimates was captured by the variation in individual admixture. Distributions of uncertainty were estimated by first calculating bootstrap 95% confidence intervals for each annual collection by sampling 10,000 times with replacement from the individual admixture data, then calculating the average admixture for each of the 10,000 samples. Note that these measures of uncertainty were different for each genetic assignment method and were not directly comparable.

After defining distributions of uncertainty in mixture proportions for each annual collection for each assignment method, we then performed the resampling statistical analysis to gauge the effect of the uncertainty on our trend analysis. We drew one sample from the distribution for each year 10,000 times with replacement, which was performed separately for the smolt and adult datasets and for each assignment method. We then ran the beta regression model for each of the 10,000 samples.

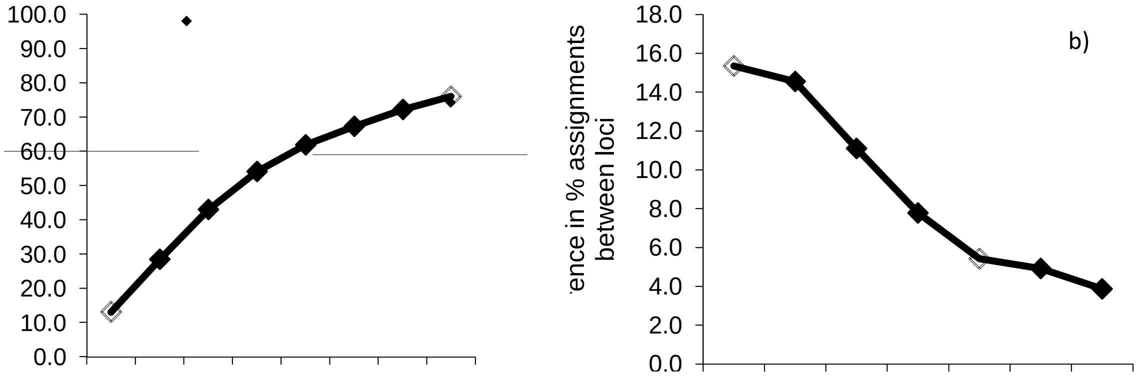

Figure S1. The effect of missing genetic data on GENECLASS2 individual assignments. Allelic data were randomly dropped from one locus per individual from all individuals with complete genotypes and reanalyzed with GENECLASS2. Panel a) shows the percent of samples with a confident assignment to either hatchery or wild versus the number of loci. Panel b) shows the difference in the percent confident assignments between numbers of loci. Increasing the number of genotyped loci above 5 did little to increase the number of individuals with a confident assignment.

Table S2. Parameter estimates from logistic regression analyses of time series data (*P*-values in parentheses) and resampling analysis.

		Beta Regression		Resampling Analysis		
		Smolts	Adults	fraction of 10,000 samples with:	Smolts	Adults
Individual	Slope	-0.0316	-0.0989	slope < 0	0.9999	0.9999
Assignment		(0.028505)	(<0.0001)	$P < 0.05$	0.5150	0.4864
(GENECLASS2)	Intercept	0.41	1.37	Intercept		
		(0.000155)	(<<0.0001)	$P < 0.05$	0.9936	0.9781
Mixture	Slope	-0.0545	-0.1150	slope < 0	0.9998	0.9739
Analysis		(0.0213)	(0.0205)	$P < 0.05$	0.5505	0.5527
(ONCOR)	Intercept	1.26	2.40	Intercept		
		(<<0.0001)	(<<0.0001)	$P < 0.05$	1.0000	0.9984
Mixture	Slope	-0.07472	-0.1149	slope < 0	1.0000	0.9696
Analysis		(0.0453)	(0.1)	$P < 0.05$	0.4594	0.3873
(BAYES)	Intercept	1.82	2.80	Intercept		
		(<<0.0001)	(<<0.0001)	$P < 0.05$	1.0000	0.9996
Admixture	Slope	-0.03374	-0.0518	slope < 0	1.0000	0.9234
Analysis		(0.0386)	(0.0573)	$P < 0.05$	0.4983	0.4491
(STRUCTURE)	Intercept	0.91	1.58	Intercept		
		(<<0.0001)	(<<0.0001)	$P < 0.05$	1.0000	0.9985

Table S3. Parameter estimates from logistic regression analyses of time series data (*P*-values in parentheses) and resampling analysis excluding early data for smolts (sample year 1998) and adults (sample years 1999, 2000).

		Logistic Regression		Resampling Analysis		
		Smolts	Adults	fraction of 10,000 samples with:	Smolts	Adults
Individual	Slope	-0.0357	-0.1041	slope < 0	0.9999	0.9998
Assignment		(0.034649)	(0.00244)	*P* < 0.05	0.4864	0.9508
(GENECLASS2)	Intercept	0.45	1.41	Intercept		
		(0.000746)	(<<0.0001)	*P* < 0.05	0.9781	0.9995
Mixture	Slope	-0.05435	-0.21786	slope < 0	0.999	0.9983
Analysis		(0.0505)	(0.000102)	*P* < 0.05	0.3759	0.8464
(ONCOR)	Intercept	1.25651	3.32	Intercept		
		(<<0.0001)	(<<0.0001)	*P* < 0.05	1.0000	0.9996
Mixture	Slope	-0.06191	-0.2582	slope < 0	0.9999	0.9995
Analysis		(0.142)	(0.00218)	*P* < 0.05	0.0935	0.8140
(BAYES)	Intercept	1.70	4.12	Intercept		
		(<<0.0001)	(<<0.0001)	*P* < 0.05	1.0000	1.0000
Admixture	Slope	-0.03226	-0.1009	slope < 0	1.0000	1.0000
Analysis		(0.0934)	(0.00207)	*P* < 0.05	0.2106	0.8485
(STRUCTURE)	Intercept	0.90	2.00	Intercept		
		(<<0.0001)	(<<0.0001)	*P* < 0.05	1.0000	1.0000

Table S4. Results of model selection analysis. *P*-values in bold type were significant after correcting for multiple tests using false discovery rate. *P*-values in italic type were significant before correcting for multiple tests.

Life History	Class	Model	Model Rank	AIC_c	ΔAIC_c	Coeff. 1	*P* -value 1	Coeff. 2	*P* -value 2	Coeff. 3	*P* -value 3	Coeff. 4	*P* -value 4
Smolt	Wild	sampyear	1	-16.29	0.0	-0.03855	*0.04720*						
		hatchnum	2	-14.24	2.1	-0.00025	0.22600						
		hatchprop	3	-13.13	3.2	-0.22680	0.60200						
		discharge	4	-12.95	3.3	-0.00147	0.76500						
		wildnum	5	-12.86	3.4	0.00000	0.99700						
		sampyear, discharge		-12.46	3.8	-0.04354	**0.02530**	-0.00415	0.33820				
		sampyear, hatchnum		-12.07	4.2	-0.06003	0.09500	0.00025	0.48070				
		sampyear, hatchprop		-11.90	4.4	-0.04588	*0.04860*	0.25980	0.56720				
		sampyear, wildnum		-11.66	4.6	-0.03935	*0.04360*	0.00003	0.76680				
		sampyear, discharge, hatchnum		-6.76	9.5	-0.06664	*0.05910*	-0.00425	0.31190	0.00026	0.43740		
		sampyear, discharge, hatchprop		-6.56	9.7	-0.05136	**0.02620**	-0.00419	0.32260	0.27431	0.53320		
		sampyear, discharge, wildnum		-6.23	10.1	-0.04412	**0.02390**	-0.00408	0.34590	0.00002	0.80360		
		sampyear, hatchnum, discharge, wildnum		1.78	18.1	-0.07228	*0.04760*	0.00032	0.36840	-0.00413	0.32130	0.00005	0.61240
	F1 Hybrid	discharge	1	-10.81	0.0	0.01144	0.07000						
		hatchnum	2	-8.78	2.0	0.00029	0.32950						
		discharge, hatchnum	3	-8.72	2.1	0.01324	**0.02050**	0.00041	0.10200				
		wildnum	4	-8.68	2.1	-0.00014	0.32272						
		hatchprop	5	-8.32	2.5	0.44300	0.46030						
		sampyear		-7.96	2.9	0.01226	0.69060						
		discharge, sampyear		-7.00	3.8	0.01308	*0.03930*	0.02545	0.34790				
		discharge, wildnum		-6.90	3.9	0.01100	0.07390	-0.00012	0.34700				
		discharge, hatchprop		-7.26	3.5	0.01197	*0.04470*	0.56296	0.27070				
		hatchprop, hatchprop^2		-3.65	7.2	0.41600	0.49540	-1.04710	0.82060				
		discharge, hatchnum, wildnum		-3.22	7.6	0.01272	**0.02260**	0.00040	0.10370	-0.00010	0.36000		
		discharge, hatchnum, sampyear		-2.72	8.1	0.01246	**0.03210**	0.00061	0.16950	-0.02534	0.58800		
		discharge, hatchnum, hatchprop		-2.65	8.2	0.01368	**0.01780**	0.00058	0.17610	-0.39984	0.62610		
		discharge, hatchnum, sampyear, wildnum		5.50	16.3	0.01235	**0.03060**	-0.01394	0.77450	0.00051	0.26740	-0.00009	0.43660
	Hatchery	discharge	1	-19.64	0.0	-0.00947	0.08180						
		discharge, hatchnum	2	-18.18	1.5	-0.01429	**0.00290**	-0.00057	**0.01420**				
		hatchprop	3	-17.85	1.8	-0.55210	0.32802						
		hatchnum	4	-17.86	1.8	-0.00026	0.36328						
		discharge, hatchnum, sampyear	5	-17.76	1.9	-0.01349	**0.00012**	-0.00148	**0.00000**	0.12415	**0.00033**		
		wildnum		-17.69	1.9	0.00013	0.38200						
		discharge, hatchprop		-17.02	2.6	-0.01245	**0.01217**	-0.97884	*0.03740*				
		sampyear		-17.03	2.6	0.00447	0.89432						
		discharge, wildnum		-14.25	5.4	-0.00927	0.08050	0.00011	0.39900				
		discharge, sampyear		-13.88	5.8	-0.01044	0.06660	-0.01545	0.62570				
		discharge, hatchnum, wildnum		-9.43	10.2	-0.01396	**0.00364**	-0.00055	**0.02253**	0.00005	0.62522		
		discharge, hatchnum, sampyear, wildnum		-2.85	16.8	-0.01364	**0.00011**	-0.00151	**0.00000**	0.12510	**0.00031**	-0.000024	0.75640
		discharge, hatchnum, hatchprop		-0.63	19.0	0.01373	**0.00061**	0.00109	**0.00099**	-0.87114	0.14459		

Adult	Wild	discharge	1	-6.53	0.0	0.02695	**0.00034**						
		discharge, hatchnum	2	-5.12	1.4	0.02194	**0.00088**	-0.000672	**0.01580**				
		hatchnum	3	-4.33	2.2	-0.00112	**0.00217**						
		sampyear	4	-4.28	2.3	-0.13195	**0.00320**						
		discharge, sampyear	5	-3.85	2.7	0.01962	**0.00731**	-0.07824	*0.04602*				
		discharge, hatchprop		-1.97	4.6	0.02625	**0.00025**	-0.72731	0.22485				
		discharge, wildnum		-0.54	6.0	0.02691	**0.00036**	-0.00001	0.92760				
		hatchprop		0.30	6.8	-1.21080	0.18700						
		wildnum		1.75	8.3	-0.00006	0.77770						
		discharge, hatchnum, hatchprop		2.91	9.4	0.02051	**0.00131**	-0.00107	**0.02103**	0.89630	0.30720		
		discharge, hatchnum, wildnum		3.74	10.3	0.02177	**0.00094**	-0.00068	**0.01393**	-0.000044	0.71946		
		discharge, hatchnum, sampyear		3.78	10.3	0.02096	**0.00334**	-0.00056	0.21614	-0.01848	0.75443		
		discharge, hatchnum, wildnum, sampyear		18.71	25.2	0.02126	**0.00324**	-0.00062	0.22063	-0.00003	0.80533	-0.01034	0.87778
	F1 Hybrid	sampyear	1	1.81	0.0	0.10856	*0.03830*						
		hatchnum	2	1.97	0.2	0.00104	*0.04870*						
		discharge	3	2.14	0.3	-0.01778	*0.04520*						
		hatchprop	4	4.76	2.9	-0.38132	0.39200						
		wildnum	5	5.07	3.3	0.00016	0.49200						
		sampyear, discharge		6.15	4.3	0.07986	0.14200	-0.01229	0.18000				
		sampyear, hatchnum		7.29	5.5	0.06591	0.40000	0.00056	0.46200				
		sampyear, hatchprop		7.56	5.7	0.10449	0.05140	0.47512	0.60350				
		sampyear, wildnum		7.81	6.0	0.10870	0.05310	0.00000	0.99660				
		hatchprop, hatchprop^2		10.75	8.9	-0.26778	0.73500	1.88300	0.79600				
		sampyear, discharge, hatchnum		14.55	12.7	0.03749	0.62400	-0.01201	0.17100	0.00058	0.43000		
		sampyear, discharge, hatchprop		14.93	13.1	0.07754	0.15700	-0.01193	0.18700	0.42571	0.62600		
		sampyear, discharge, wildnum		15.15	13.3	0.08004	0.16600	-0.01229	0.18000	0.00000	0.99300		
		sampyear, discharge, hatchnum, wildnum		29.46	27.6	0.02420	0.78200	-0.01194	0.17000	0.00066	0.40000	0.00006	0.76100
	Hatchery	hatchprop	1	4.72	0.0	1.44270	**0.00537**						
		hatchnum	2	6.45	1.7	0.00058	**0.03320**						
		wildnum	3	8.47	3.8	-0.00050	0.26500						
		discharge	4	8.95	4.2	-0.00345	0.51618						
		sampyear	5	9.35	4.6	0.01940	0.61300						
		hatchprop,sampyear		∞	NA	2.83909	**0.00000**	-0.07696	**0.00001**				
		hatchprop,wildnum		∞	NA	1.38468	**0.00836**	-0.00030	0.41269				
		hatchprop,discharge		∞	NA	1.34809	**0.01250**	-0.00178	0.67480				
		hatchprop,hatchnum		∞	NA	2.37090	0.05020	-0.00045	0.41000				

Table S5. Proportions of smolts of wild, unassigned and hatchery ancestry outmigrating at 1, 2 , or 3 years of age.

	Age			
Ancestry	1	2	3	Total n
Wild	0.34	0.54	0.11	78
Unassigned	0.44	0.48	0.08	63
Hatchery	0.63	0.28	0.09	140
Total n	125	128	28	281
